# Facial Analysis for Plastic Surgery in the Era of Artificial Intelligence: A Comparative Evaluation of Multimodal Large Language Models

**DOI:** 10.3390/jcm14103484

**Published:** 2025-05-16

**Authors:** Syed Ali Haider, Srinivasagam Prabha, Cesar A. Gomez-Cabello, Sahar Borna, Ariana Genovese, Maissa Trabilsy, Adekunle Elegbede, Jenny Fei Yang, Andrea Galvao, Cui Tao, Antonio Jorge Forte

**Affiliations:** 1 Division of Plastic Surgery, Mayo Clinic, Jacksonville, FL 32224, USA; 2 School of Dentistry, University Center UNICHRISTUS, Fortaleza 60190-180, Brazil; 3 Department of Artificial Intelligence and Informatics, Mayo Clinic, Jacksonville, FL 32224, USA; 4 Center for Digital Health, Mayo Clinic, Rochester, MN 55905, USA

**Keywords:** artificial intelligence, multimodal large language models, large language models, facial analysis, facial plastic surgery

## Abstract

**Background/Objectives:** Facial analysis is critical for preoperative planning in facial plastic surgery, but traditional methods can be time consuming and subjective. This study investigated the potential of Artificial Intelligence (AI) for objective and efficient facial analysis in plastic surgery, with a specific focus on Multimodal Large Language Models (MLLMs). We evaluated their ability to analyze facial skin quality, volume, symmetry, and adherence to aesthetic standards such as neoclassical facial canons and the golden ratio. **Methods:** We evaluated four MLLMs—ChatGPT-4o, ChatGPT-4, Gemini 1.5 Pro, and Claude 3.5 Sonnet—using two evaluation forms and 15 diverse facial images generated by a Generative Adversarial Network (GAN). The general analysis form evaluated qualitative skin features (texture, type, thickness, wrinkling, photoaging, and overall symmetry). The facial ratios form assessed quantitative structural proportions, including division into equal fifths, adherence to the rule of thirds, and compatibility with the golden ratio. MLLM assessments were compared with evaluations from a plastic surgeon and manual measurements of facial ratios. **Results:** The MLLMs showed promise in analyzing qualitative features, but they struggled with precise quantitative measurements of facial ratios. Mean accuracy for general analysis were ChatGPT-4o (0.61 ± 0.49), Gemini 1.5 Pro (0.60 ± 0.49), ChatGPT-4 (0.57 ± 0.50), and Claude 3.5 Sonnet (0.52 ± 0.50). In facial ratio assessments, scores were lower, with Gemini 1.5 Pro achieving the highest mean accuracy (0.39 ± 0.49). Inter-rater reliability, based on Cohen’s Kappa values, ranged from poor to high for qualitative assessments (κ > 0.7 for some questions) but was generally poor (near or below zero) for quantitative assessments. **Conclusions:** Current general purpose MLLMs are not yet ready to replace manual clinical assessments but may assist in general facial feature analysis. These findings are based on testing models not specifically trained for facial analysis and serve to raise awareness among clinicians regarding the current capabilities and inherent limitations of readily available MLLMs in this specialized domain. This limitation may stem from challenges with spatial reasoning and fine-grained detail extraction, which are inherent limitations of current MLLMs. Future research should focus on enhancing the numerical accuracy and reliability of MLLMs for broader application in plastic surgery, potentially through improved training methods and integration with other AI technologies such as specialized computer vision algorithms for precise landmark detection and measurement.

## 1. Introduction

The face holds a unique position as the most prominent and expressive feature of the human body, making it central to individual identity and social perception [1]. Artists and scholars throughout history have been intrigued by the intricacies of facial aesthetics, seeking to define the ideal proportions and features that contribute to perceived beauty [2]. In today’s digital age, with the pervasive influence of social media and visual platforms, individuals are increasingly attuned to subtle nuances in facial features and aesthetics [3]. This heightened awareness further emphasizes the importance of accurate and comprehensive facial analysis in plastic surgery [4].

Facial analysis plays a pivotal role in facial plastic surgery, serving as the cornerstone for effective preoperative planning [5]. This crucial step is undertaken before any major facial procedure, encompassing both aesthetic enhancements and the rectification of congenital anomalies [6,7]. The accuracy and efficiency of facial analysis are of paramount importance, as they directly influence the achievement of optimal surgical outcomes and patient satisfaction [8].

Conventional approaches to facial analysis predominantly rely on anthropometric measurements, 2D imaging and subjective assessments by surgeons [9,10]. While these methods establish a foundational approach, they suffer from inherent limitations. The reliance on manual processes leads to time-consuming procedures and subjective interpretations, which can introduce inconsistencies [11]. These factors may compromise the accuracy and efficiency of preoperative assessments, ultimately affecting patient satisfaction. Existing methods can also struggle to reflect the subtle aesthetic ideals of different demographic groups, leading to potentially suboptimal outcomes. The need for more objective, comprehensive, and patient-specific facial analysis techniques is therefore paramount.

Artificial Intelligence (AI) and Multimodal Large Language Models (MLLMs) are driving a paradigm shift across various fields within healthcare, particularly in the domain of medical image analysis. [12,13] These models possess a remarkable ability to process and analyze both textual and visual information, enabling advancements in objective facial analysis across diverse medical disciplines. Notably, AI-driven facial analysis is increasingly employed in orthodontics, forensic medicine, and general medical imaging. [14,15,16,17,18]. Given the critical role of precise facial analysis in plastic surgery, we sought to evaluate the potential of MLLMs, leveraging their multimodal capabilities, to facilitate this process. Unlike traditional computer vision techniques, which often rely on predefined features, MLLMs can learn complex patterns and relationships directly from data, potentially capturing subtle aesthetic cues that may be missed by human observers or simpler algorithms [19]. This capability is crucial in quantifying properties of the face, such as harmony, that are traditionally considered subjective. Furthermore, the potential biases and limitations of MLLMs in this specific context require thorough investigation to ensure responsible and ethical implementation [20,21,22].

This research is novel in its comparison of most popular commercially available MLLMs to the specific challenges of facial analysis in plastic surgery. The application of MLLMs to comprehensive facial assessment, including both objective measurements and subjective aesthetic evaluations, remains largely unexplored, notably with no prior studies comparatively evaluating multiple MLLMs or comparing their assessments to those of plastic surgeons. This study builds upon previous work in computer vision and extends it by incorporating the contextual understanding and multimodal capabilities of MLLMs.

We conducted a comparative assessment of MLLM-generated facial analyses against assessments by experienced plastic surgeons, focusing on qualitative assessments. We further assessed MLLM performance on quantitative measurements against established neoclassical canons, including the golden ratio. Neoclassical canons are a set of ideal facial proportions derived from classical art [23], while the golden ratio (approximately 1.618) is a mathematical proportion often found in nature and art, believed to be inherently aesthetically pleasing [24]. The potential implications of this research are significant, offering the prospect of more precise surgical planning, improved patient–surgeon communication, more objective outcome assessment, and the potential for personalized aesthetic ideals to be better understood and implemented. This could ultimately lead to enhanced patient satisfaction and a deeper understanding of the complex interplay of factors that contribute to facial aesthetics.

## 2. Methods

**Figure 1:**
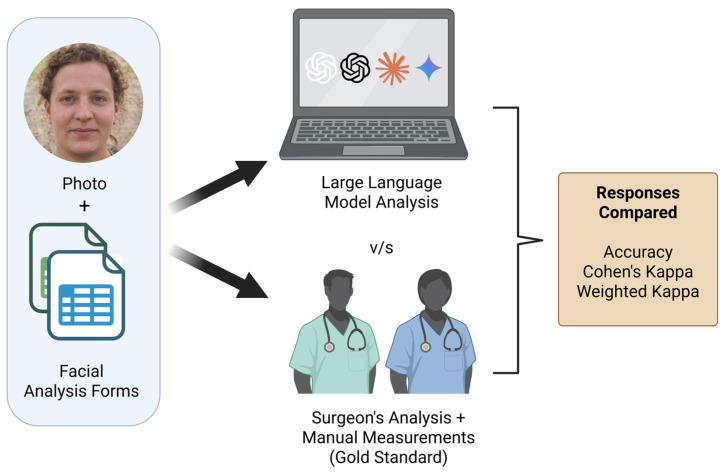
Study workflow. Facial images and standardized forms were presented to four MLLMs. Their responses were compared to a gold standard consisting of evaluations by plastic surgeons (for qualitative analysis) and manual measurements (for quantitative analysis). Performance was assessed using accuracy, Cohen’s Kappa, and Weighted Kappa.

We evaluated the ability of four multimodal large language models (MLLMs)—ChatGPT-4 (ChatGPT; OpenAI, San Francisco, CA, USA, Paid), ChatGPT-4o (ChatGPT; OpenAI, San Francisco, CA, USA, Paid), Gemini 1.5 Pro (Gemini; Google DeepMind, Mountain View, CA, USA, Paid), and Claude 3.5 Sonnet (Claude; Anthropic, San Francisco, CA, USA, Paid)—to conduct facial analysis for plastic surgery preoperative assessment. The MLLMs were tested using two standardized forms and a dataset of frontal facial photographs. Their responses were compared to expert assessments: qualitative answers were benchmarked against evaluations from experienced plastic surgeons, and quantitative measurements were validated against manual calculations (Figure 1). The evaluation utilized the base, generalist MLLMs without specific fine-tuning or Retrieval-Augmented Generation (RAG) on specialized facial analysis datasets, reflecting their readily available capabilities.

### 2.1. Image Dataset

**Figure 2:**
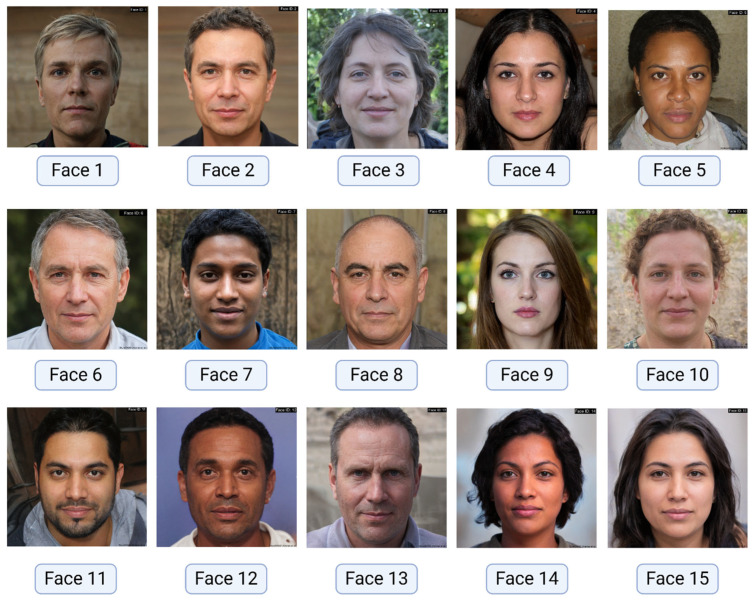
Facial images dataset comprising 15 frontal facial photographs.

A dataset of 15 diverse, AI-generated facial images was created using “This Person Does Not Exist” (URL: www.thispersondoesnotexist.com, accessed on 28 June 2024), a Random Face Generator created by Philip Wang [25,26]. This website employs advanced Generative Adversarial Network (GAN) technology to produce photorealistic images of non-existent individuals, ensuring a controlled and privacy-preserving dataset. The images were selected to represent a range of ages, ethnicities, and facial features to ensure comprehensive analysis (Figure 2). The use of AI-generated images ensured a controlled and privacy-preserving dataset, eliminating concerns related to Protected Health Information (PHI) inherent in using real patient photographs.

### 2.2. Assessment Forms

Two structured forms were developed to standardize responses and facilitate comparison with expert assessments, based on the current literature on facial analysis and professional standards in plastic surgery [27]:

- Qualitative Facial Analysis Form: This form comprised 14 questions assessing various aspects of facial features relevant to plastic surgery, inquiries on key aspects such as skin type and texture identification, the assessment of facial symmetry, and the evaluation of volume and fat distribution. The full form is provided in Table 1.
- Quantitative Ratios Form: This form comprised 15 questions, centering on neoclassical canons and golden ratio calculations to assess the MLLMs’ ability to measure facial proportions and feature relationships. This form specifically focused on evaluating key ratios such as the vertical fifths of the face, the equality of facial thirds, and the relationship between interocular distance and nose width. The full form is provided in Table 2.

### 2.3. Prompting Protocol

**Figure 3:**
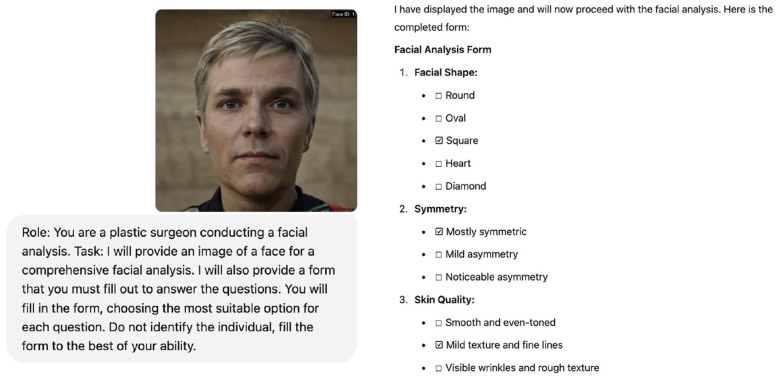
Demonstration of facial analysis using ChatGPT-4o.

Effective prompt engineering was crucial for eliciting relevant and standardized responses from the MLLMs. Initial prompts requesting general facial analysis led to the MLLMs declining to respond due to potential identification risks. Therefore, subsequent prompts were refined to specify the context as a plastic surgery study. These refined prompts explicitly instructed the MLLMs to focus solely on facial features and proportions, avoiding individual identification. The detailed assessment criteria and questions were presented directly within the prompt text itself, structured as two comprehensive forms (Table 1 and Table 2), rather than using simple free-text queries or external files. For each facial image, Form 1 was presented as the prompt in a new, independent chat session for each MLLM. Subsequently, Form 2 was presented as the prompt in a separate, new independent chat session for the same image and MLLM to ensure responses were not influenced by previous image analyses within the same conversation history. Each MLLM was presented with each form, one at a time, along with the corresponding facial image (Figure 3).

### 2.4. Validation Process

To establish a benchmark for MLLM performance, a validation process was implemented using expert and manual methods:

- Expert Validation (Qualitative Data): Two experienced plastic surgeons independently analyzed each image and completed the qualitative facial analysis form. Serving as the control group, their independent assessments were compared to the MLLM responses. Discrepancies between the two surgeons’ initial evaluations were resolved by consulting a third plastic surgeon, whose evaluation served as the final, gold standard for the form.
- Manual Measurement Validation (Quantitative Data): For the facial ratios form, two researchers independently performed manual measurements of facial ratios in each image using ImageJ Software (v1.54), the standardized measurement technique. The mean of these measurements served as the gold standard for validating the MLLMs’ quantitative responses.

### 2.5. Data Analysis

The accuracy and consistency of MLLM outputs were evaluated using the following metrics:

- Accuracy: Calculated as the percentage of correct classifications for both categorical and ordinal data.
- Cohen’s Kappa: Used to assess inter-rater reliability between MLLMs and plastic surgeons/manual measurements for categorical data.
- Weighted Cohen’s Kappa: Employed to account for the degree of disagreement in ordered categories for ordinal data.

### 2.6. Statistical Analysis

The Intraclass Correlation Coefficient (ICC) was used to assess consistency between MLLMs and plastic surgeons and between MLLMs and calculated facial ratios. Subgroup and error analyses were conducted to investigate performance variations and identify patterns in MLLM performance.

## 3. Results

This section presents the descriptive statistics for each MLLM’s overall performance on both the forms. Detailed question-level performance data for descriptive statistics is available in Supplementary Tables S1 and S2. Inter-rater reliability analyses are available in Supplementary Materials Table S3.

### 3.1. The Qualitative Form (General Facial Analysis)

An overview of the ranked performance metrics resulting from the qualitative analysis is provided in Table 3. 

#### 3.1.1. Inter-Model Performance in Qualitative Analysis

ChatGPT-4o achieved the highest mean accuracy (0.61, SD = 0.49), closely followed by Gemini 1.5 Pro (0.60, SD = 0.49). Claude 3.5 Sonnet exhibited the lowest mean accuracy (0.52, SD = 0.50), while ChatGPT-4 demonstrated an accuracy of 0.57 (SD = 0.50). The standard deviations for all models were relatively high (ranging from 0.49 to 0.50), indicating considerable variability in performance across the different questions and faces.

**Figure 4:**
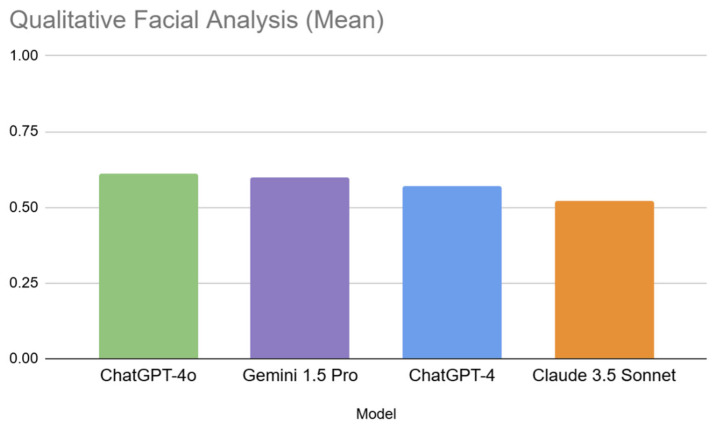
Bar chart demonstrating MLLM performance across qualitative facial analysis.

A one-way Analysis of Variance (ANOVA) was conducted to determine if the observed differences in mean accuracy between the LLMs were statistically significant. The results revealed no statistically significant difference between the models’ performance (F(3, 56) = 1.36, *p* = 0.255). The effect size, calculated as eta-squared (η^2^), was 0.005, suggesting that only 0.5% of the variance in accuracy was attributable to the different MLLMs. This indicates a very small practical difference between the models. Figure 4 visually demonstrates the MLLM’s performance across this analysis.

#### 3.1.2. Question Difficulty Analysis for Qualitative Form

Substantial variability was observed in the difficulty levels of the 14 qualitative questions. The most challenging question was Question 6 (“Using the Glogau classification, determine the degree of photoaging”), with a mean accuracy of only 0.020. In stark contrast, the easiest question was Question 3 (“Volume and Fat Distribution in Cheeks”), achieving a mean accuracy of 0.82. Other questions with high accuracy included Questions 11 (“Extent of Jowls”) and 12 (“Visibility of Platysmal Bands”), both at 0.73. Questions such as Question 9 (“Assess the symmetry of the nasolabial folds”) with a mean accuracy of 0.27, demonstrated moderate difficulty.

A one-way ANOVA was performed to statistically assess the differences in mean accuracy across the questions. The results indicated a highly significant difference in performance across the different questions (F(13, 42) = 12.91, *p* < 0.001). The effect size, eta-squared (η^2^), was 0.17, indicating that 16.9% of the variance in accuracy was explained by the question type. This represents a moderate to large effect, confirming that the type of question significantly impacted the accuracy of the MLLMs.

#### 3.1.3. Facial Image Difficulty Analysis in Qualitative Form

In addition to MLLM and question-specific performance, we analyzed the variability in accuracy across the 15 different faces presented to the models. The mean accuracy for each face, averaged across all MLLMs and questions, is reported. Face 8 exhibited the lowest mean accuracy (0.21, SD = 0.41), indicating that this face was the most challenging for the MLLMs to analyze qualitatively. Conversely, Face 4 had the highest mean accuracy (0.80, SD = 0.40), suggesting it was the easiest for the models to assess. Other faces with relatively high accuracy included Face 2 (0.77, SD = 0.43) and Face 9 (0.71, SD = 0.46), while faces such as Face 6 (0.36, SD = 0.48) and Face 3 (0.45, SD = 0.50) presented greater difficulty.

A one-way ANOVA was conducted to determine if the differences in accuracy across faces were statistically significant. The analysis revealed a highly significant difference in performance (F(14, 825) = 7.041, *p* < 0.001). The effect size, as measured by eta-squared (η^2^), was 0.107. This indicates that 10.7% of the variance in accuracy was attributable to the specific face being analyzed, representing a moderate effect. This finding highlights that older vs. younger facial characteristics, independent of the question being asked, significantly influenced the MLLMs’ ability to provide accurate qualitative assessments.

### 3.2. The Quantitative Form (Facial Ratios and Adherence to Golden Ratio)

#### 3.2.1. Inter-Model Performance in Quantitative Analysis

The quantitative analysis assessed the MLLMs’ ability to determine key facial ratios, and proportions, across the 15 images. Accuracy rates were substantially lower than for qualitative analysis, indicating challenges in quantitative facial analysis. Overall, the mean accuracy across all models and questions was 0.35 (SD = 0.48). Gemini 1.5 Pro achieved the highest mean accuracy (0.39, SD = 0.489), followed by Claude 3.5 Sonnet (0.37, SD = 0.49). Both ChatGPT-4 and ChatGPT-4o had a mean accuracy of 0.32 (SD = 0.47). Similarly to the qualitative form, a high standard deviation was obtained, indicating variability.

A one-way ANOVA was conducted to compare the performance of the four MLLMs. No statistically significant difference was found between the models (F(3, 896) = 1.33, *p* = 0.264). The effect size, eta-squared (η^2^), was 0.004, representing a very small effect, and only 0.4% of variability was explained.

#### 3.2.2. Question Difficulty Analysis for Quantitative Form

Significant variability was observed in the accuracy of responses across the 15 quantitative questions. Question 10 (“The length of the face is 1.618 times the width of the face?”) had the lowest accuracy, with a mean of 0.00 across all MLLMs, indicating that none of the models could correctly assess this statement. Question 11 (“Ear length to nose width ratio”) and Question 15 (“Lips-chin distance to nose width”) were also particularly challenging, with means of 0.02. Conversely, Question 9 (“Is the width of the face at the malar level equal to the distance from the brows to the menton?”) was the easiest, with a mean accuracy of 0.75 (SD = 0.44). Other questions with relatively high accuracies included Question 8 (“Can the lower face be divided into equal thirds?”) at 0.70 (SD = 0.46) and Question 5 (“Is the interocular distance equal to the width of one eye (right or left eye fissure width)?”) at 0.53 (SD = 0.50).

A one-way ANOVA was conducted to statistically evaluate these differences. A highly significant difference in accuracy was found across the questions (F(14, 885) = 21.82, *p* < 0.001). The effect size, eta-squared (η^2^), was 0.26, indicating that 25.7% of the variance in accuracy was explained by the question type. This is considered a large effect.

Post hoc analysis using Tukey’s HSD revealed multiple significant pairwise differences between questions. Specifically, Questions 10, 11, and 15 (all with very low accuracy) were significantly different from Questions 8 and 9 (the highest accuracy questions).

#### 3.2.3. Facial Image Difficulty Analysis in Quantitative Form

Analysis of face-specific performance on the quantitative questions revealed variations in accuracy across the 15 images. Face 8 achieved the highest mean accuracy (0.50, SD = 0.50), closely followed by Face 10 with a mean accuracy of (0.47, SD = 0.50). Conversely, Face 4, Face 14, and Face 15 had the lowest mean accuracy, each at 0.25, with standard deviations of 0.44, indicating they were the most challenging for the MLLMs to analyze quantitatively.

A one-way ANOVA was performed to assess the statistical significance of these differences. The analysis revealed a statistically significant difference in accuracy across the different faces (F(14, 885) = 1.76, *p* = 0.040). Although statistically significant, the effect size was small. The eta-squared (η^2^) value was 0.027, indicating that only 2.7% of the variance in accuracy was attributable to the specific face.

Post hoc analysis using Tukey’s HSD did not reveal any significant pairwise differences between specific faces. All faces fell within a single homogeneous subset, suggesting that while there was an overall significant difference between faces, the differences between individual faces were not large enough to be statistically distinguishable at the alpha = 0.05 level. This indicates there are, overall, differences across faces, the individual face differences were subtle in the quantitative assessment.

**Figure 5:**
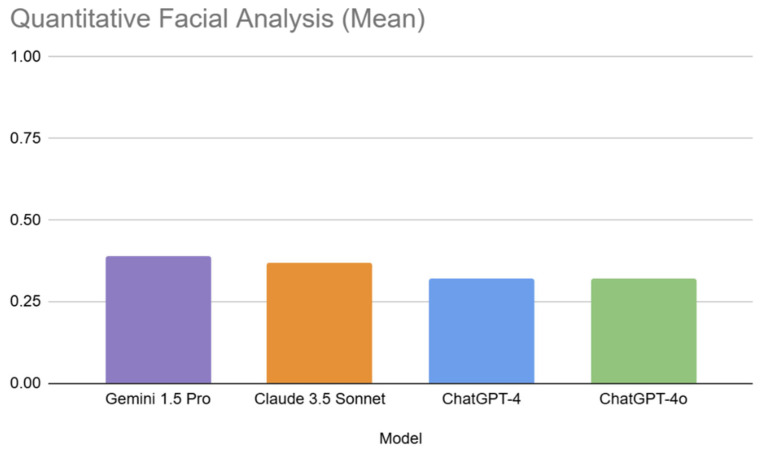
Bar chart demonstrating MLLM performance across quantitative facial analysis.

Overview of the ranked performance metrics for the quantitative form are presented in Table 4 and MLLM performance in Figure 5.

### 3.3. Inter-Rater Reliability of MLLMs

Cohen’s and weighted Kappa coefficients were calculated to assess agreement between MLLM responses and expert human ratings (Supplemental Table S3).

- Qualitative Assessment: Reliability varied considerably. High agreement (κ > 0.7) was observed for some models on questions like “Volume and Fat Distribution in Cheeks”, “Presence of Rhytids”, and “Overall balance and harmony of the face”. However, other questions, notably “Visibility of Platysmal Bands”, and “Fat Herniation of Eyelid” showed consistently poor agreement across all models.
- Quantitative Assessment: Inter-rater reliability was substantially lower than in the qualitative assessment. Most Kappa values were near or below zero, indicating poor agreement between the MLLMs and manual measurements. No question reached substantial levels of agreement.

While some MLLMs demonstrated strong agreement with human raters on select qualitative features, overall reliability was inconsistent and particularly low for quantitative assessments, highlighting the importance of question type.

## 4. Discussion

This study aimed to investigate the capabilities and limitations of currently available, general-purpose MLLMs accessed via structured prompting, rather than specifically trained or fine-tuned AI models, for comprehensive facial analysis in plastic surgery. While MLLMs possess significant potential for assisting in facial analysis, their capabilities are currently limited by task complexity and a lack of domain-specific knowledge. Our primary findings demonstrated that, while MLLM performance did not differ significantly between models, performance varied dramatically across different question types and, to a lesser extent, across different faces. Notably, for qualitative assessments, the MLLMs demonstrated a tendency to perform best when analyzing younger faces. Furthermore, inter-rater reliability between MLLMs and human experts was highly variable, particularly for quantitative assessments.

A notable strength of this study is its systematic approach to evaluating advanced MLLMs against rigorous benchmarks—expert surgeons’ qualitative evaluations and precise manual quantitative measurements. This comprehensive validation method provides robust insight into the actual clinical utility of these models, enabling a more nuanced understanding of their applicability in clinical practice. Furthermore, the inclusion of diverse facial images with various skin tones and age groups enhanced the generalizability of the results.

However, the study also highlighted significant variability in model performance. Quantitative assessments, particularly involving adherence to neoclassical facial ratios and golden ratio standards, were notably difficult for all models tested. The variability and relatively low inter-rater reliability observed suggest that current MLLMs are less effective in accurately capturing subtle proportional aspects of facial morphology.

AI-driven facial analysis is increasingly being utilized across healthcare for diverse purposes, including health monitoring, screening, rare disease diagnosis, facial age assessment, and supporting clinical decisions [14,28,29]. Recent advancements in machine learning and deep learning have significantly impacted person detection, tracking, identification, and face recognition technologies [30]. Our findings align with several studies utilizing AI and computer vision techniques for facial analysis in clinical and medical contexts. AI tools like the F4CE app have shown high accuracy in specific facial measurement tasks [31]. Our findings indicate that MLLMs currently struggle with the level of precision required for detailed quantitative analysis, a discrepancy also highlighted in the evaluation of ChatGPT by Ali et al. [32] Their study utilized a custom ChatGPT model for aesthetic evaluations in minimally invasive facial procedures and also assessed facial features based on objective parameters such as the golden ratio and symmetry. The authors found that skin evaluations and overall harmony showed statistically significant improvements, a finding that also resonates with the qualitative analysis of our study. AI models frequently demonstrate strong qualitative capabilities, for instance, in emotion recognition, facial palsy assessment, and evaluation of facial attractiveness [15,33,34,35,36]. Studies employing deep learning for facial palsy classification have similarly found AI models to accurately classify severity and distinguish between different forms of palsy with high consistency [33]. Utilizing AI assessments can provide consistent and objective evaluations, especially beneficial in scenarios where human assessments might vary due to subjective biases or observer fatigue.

Despite the alignment on qualitative capabilities, important divergences emerged in quantitative facial analyses. Unlike studies focused solely on classification tasks (e.g., emotion recognition or palsy severity grading), which generally report high accuracies, our findings highlight significant difficulties in precise measurement and proportional analysis tasks using MLLMs. While the recent literature has successfully leveraged convolutional neural networks (CNNs) and LLMs to achieve high accuracy in cephalometric landmark detection and proportional measurements [37,38], the use of multimodal language-based models (like ChatGPT or Gemini) for similar detailed quantitative tasks appears less effective. Salinas et al. demonstrated the effectiveness of employing morphometrics based on facial landmarks, analyzed through an AI platform, to provide valuable, ethnicity-specific data for contemporary facial analysis relevant to plastic surgery [39]. This discrepancy likely stems from the generalist nature of MLLMs compared to purpose-built, domain-specific models that benefit from specialized training on facial measurement datasets. Inspired by the growing use of neural networks in orthodontics to automate anatomical landmark detection in cephalometric analysis, integrating similar specialized computer vision components into LLMs holds the potential to address the challenges our study faced with spatial reasoning and fine-grained detail extraction for quantitative assessments [40]. Unlike specialized computer vision models specifically trained for precise anthropometric tasks, the generalist architecture and training of MLLMs prioritize broader pattern recognition and conceptual understanding over the granular spatial analysis required for accurate quantitative measurements. Reflecting the increasing use of AI in aesthetic dentistry and orthodontics, Maniega-Mañes et al. trained a neural network that demonstrated high precision and efficiency, significantly outperforming conventional methods like Digital Smile Design (DSD) for tasks such as computerized facial landmark identification [41]. This success highlights that task-specific AI models, trained appropriately, may overcome precision challenges currently faced by more generalized models in specialized clinical applications.

This contrast suggests that while current generalist MLLMs perform well in qualitative description and classification, their capability for precise numerical and proportional analysis remains limited compared to specialist AI programs like computer vision models tailored explicitly for facial landmark identification. Our findings underscore both the potential and limitations of integrating MLLMs into clinical facial assessment workflows. Their qualitative strengths demonstrate promise for preliminary consultations, patient education, and improving surgeon–patient communication by objectively highlighting facial symmetry and proportional concerns.

However, limitations in quantitative precision suggest that MLLMs should currently serve as adjunctive rather than primary diagnostic tools. Their utility is maximized when combined with specialized computer vision models capable of precise measurements, emphasizing the value of hybrid approaches in clinical practice.

### 4.1. Limitations

Several important limitations must be considered when interpreting our findings. First, the use of AI-generated facial images, while ensuring privacy, may not fully represent the complexity and variability of real patient photographs. Features such as scars, complex asymmetries, specific pathological conditions, or subtle nuances of age-related and previous surgical alterations commonly encountered in clinical practice were likely underrepresented, potentially limiting the evaluation of MLLM performance on the full spectrum of clinical presentations.

Second, the prompt engineering process revealed significant constraints in how general purpose MLLMs can be directed to perform facial analysis. Initial attempts at general facial assessment were declined by the models due to potential privacy concerns, necessitating specific contextual framing. This highlights important ethical considerations in deploying these technologies in clinical settings.

Third, the reliance on three experienced plastic surgeons for expert validation, while providing a valuable benchmark, may introduce a degree of subjectivity inherent in human assessments. Expanding the pool of expert validators could enhance the objectivity of the findings.

Furthermore, our study assessed MLLMs at a single point in time, yet these models undergo frequent updates that may significantly alter their capabilities. The rapid evolution of this technology suggests that performance benchmarks may have limited temporal validity. The MLLMs evaluated were general purpose, not specifically trained AI environments utilizing domain-specific datasets and architectures for facial analysis, potentially explaining their limited performance on specialized tasks like the Glogau classification. Fine-tined models, Retrieval Augmented Generation (RAG), specialized computer vision algorithms would likely achieve higher levels of accuracy. This raises questions about whether general-purpose MLLMs can effectively serve specialized clinical domains without domain-specific optimization.

The study also had limitations related to the scope of facial analysis. It focused solely on 2D frontal facial analysis and did not include assessments from the lateral (profile) view, or even a 3D view, which is an important aspect of comprehensive facial analysis in plastic surgery. Due to the technical limitations of LLMs and challenges in calibrating pixel-to-millimeter conversion, the study primarily focused on facial proportions rather than absolute lengths. The scope of facial analysis in this study was defined by the questions included in the standardized forms. A more open-ended evaluation of MLLM capabilities, without the constraints of these specific questions, might reveal additional strengths or weaknesses that were not captured.

Finally, as with any AI technology, MLLMs may be susceptible to biases related to race, gender, culture, and age [42,43]. These potential biases should be carefully investigated and mitigated to ensure responsible and ethical implementation.

### 4.2. Ethical Considerations

Deploying AI technologies, such as MLLMs, in clinical facial analysis necessitates careful consideration of ethical issues and potential implications, reflecting insights from recent discourse on AI ethics. Given the sensitive nature of facial imaging data, ensuring patient confidentiality and data protection is paramount. The recent literature underscores concerns regarding unauthorized data access, misuse, or breaches [44]. Implementation of stringent data anonymization, secure storage solutions, and transparent data management practices must be prioritized to maintain patient trust and comply with regulatory standards [45].

It is important to acknowledge the evolving understanding of facial aesthetics. Contemporary research suggests that while the golden ratio and neoclassical canons are appealing, they are not definitive formulas for beauty [46,47]. Beauty is influenced by a complex interplay of biological factors, psychological perceptions, and cultural norms. Modern aestheticians argue against a universal mathematical formula for beauty, recognizing the importance of cultural and ethnic variations in facial features [48,49].

AI models trained on biased or non-representative datasets risk perpetuating systematic disparities, potentially disadvantaging underrepresented groups [50]. Careful attention to dataset representativeness and systematic auditing of AI outputs for fairness across diverse populations is essential. Future model training and validation should explicitly address demographic equity to prevent inadvertent bias in clinical outcomes.

Sophisticated AI systems, including MLLMs, act like a “black-box”, meaning their internal workings and the exact reasons behind their outputs are often opaque and difficult for humans to fully understand or interpret [13]. This poses ethical challenges, limiting clinician understanding and patient trust. Efforts should be directed towards developing explainable AI (XAI) approaches and chain-of-thought prompting that clearly communicate model reasoning processes for their decisions [51,52]. Transparent model explanations will enable informed clinical decision-making and patient consent.

As AI technologies increasingly inform patient decisions, there is a risk of creating unrealistic expectations. Inaccurate measurements, if presented as definitive, can lead to unrealistic patient expectations, potentially resulting in dissatisfaction or emotional distress postoperatively [53]. Additionally, inaccuracies in quantitative facial assessments could lead clinicians to incorrect diagnostic conclusions or suboptimal treatment planning, directly impacting patient safety and clinical outcomes. Therefore, AI-generated outputs must be transparently communicated as supportive rather than conclusive assessments.

### 4.3. Future Research Directions

Enhancing the accuracy and clinical utility of MLLMs for facial analysis requires targeted strategies informed by recent technological advancements. To better assess the generalizability and robustness of MLLMs for this application, future studies should evaluate their performance on larger and more diverse datasets that include real patient images representing a wider range of demographic characteristics and clinical conditions. Implementing these evaluations within controlled, ‘sandboxed’ clinical prompting environments would be crucial to mitigate challenges related to data privacy and ensure standardized, safe interaction during patient-specific analysis. Additionally, incorporating multi-view or 3D facial analysis, including profile and oblique views, would provide a more comprehensive evaluation of MLLMs’ capabilities in a setting that more closely mirrors clinical practice. By correlating AI-generated preoperative assessments with postoperative results and patient satisfaction metrics, researchers can refine AI models based on real-world performance and further enhance their predictive accuracy.

Future studies should explore the development of real-time interactive AI tools that allow surgeons to dynamically manipulate and assess facial proportions during consultations. By integrating real-time feedback into MLLM interfaces, clinicians could adjust proposed surgical plans based on AI-assisted symmetry and proportionality assessments, enhancing patient education and shared decision-making.

Implementing attention mechanisms and visual heatmaps to show which features influence MLLM-based decisions could improve clinician trust and facilitate safer AI-assisted assessments.

More research should investigate the feasibility of personalized AI frameworks tailored to individual patients. Training AI models using patient-specific datasets, including past surgical history, anatomical variability, and aesthetic preferences, could lead to more precise and personalized preoperative recommendations [54]. Personalized models may improve the accuracy of patient-specific facial assessments and optimize surgical planning.

The incorporation of MLLMs into mobile applications and wearable technologies, such as smartphone-based 3D facial scanning tools, the integration of fully automated methods for landmarking, and facial segmentation on 3D photographs, represents a promising future direction [55]. By enabling remote facial assessments, such technologies could expand access to expert-level consultations, particularly in regions with limited access to specialized plastic surgeons [31]. Research should explore the reliability and clinical validity of AI-driven facial assessments conducted via mobile platforms.

## 5. Conclusions

Our evaluation highlights that while current general-purpose MLLMs, without domain-specific training or fine-tuning, can offer some assistance in general facial feature analysis when guided by structured prompts, they are currently insufficient to replace manual clinical assessments, especially for precise quantitative measurements. The methodology employed was designed to evaluate the performance of these off-the-shelf models and inform the clinical community about their present utility and limitations in the context of plastic surgery facial analysis. The observed performance variability across different facial features and measurements suggests that these technologies currently offer complementary rather than replacement capabilities for expert clinical assessment.

As MLLMs continue to evolve rapidly, their potential applications in plastic surgery merit ongoing investigation, with particular attention to domain-specific optimization, clinical integration pathways, and careful validation across diverse patient populations. The integration of these emerging AI systems into plastic surgery practice should proceed with appropriate recognition of both their capabilities and limitations to ensure they enhance rather than compromise patient care.

## Figures and Tables

**Table 1:** Qualitative facial analysis form with options provided to the MLLMs.

#	Question	Options
1	Identify the skin type	Normal, Oily, Dry
2	Comment on the skin texture	Smooth, Rough, Presence of acne, Scarred
3	Volume and Fat Distribution in Cheeks	Well-distributed, youthful volume, Slight sagging or descent, Moderate sagging or descent, Severe sagging or loss of volume
4	How would you rate the overall tone of the skin?	Excellent, Good, Fair, Poor
5	Presence of Rhytids (Wrinkles) in the face:	No visible wrinkles, Shallow wrinkles, Moderate wrinkles, Deep wrinkles
6	Using the Glogau classification, determine the degree of photoaging	Glogau Type I: Mild wrinkles, no keratoses, early photoaging, Glogau Type II: Moderate wrinkles, early actinic keratoses, early to moderate photoaging, Glogau Type III: Advanced wrinkles, actinic keratoses, advanced photoaging, Glogau Type IV: Severe wrinkles, actinic keratoses and skin cancers, severe photoaging
7	Assess the Overall balance and harmony of the face.	Excellent, Good, Fair, Poor
8	Overall Proportions and Symmetry of the Nose	Proportions and symmetry ideal, Slight disproportion or asymmetry, Moderate disproportion or asymmetry, Severe disproportion or asymmetry
9	Assess the symmetry of the nasolabial folds	Perfectly symmetrical, Slightly asymmetrical, Moderately asymmetrical, Highly asymmetrical
10	Assess the depth of the nasolabial folds	Very shallow, Moderately shallow, Moderately deep, Very deep
11	Extent of Jowls	No jowls, Slight jowls, Moderate jowls, Severe jowls affecting facial contour
12	Visibility of Platysmal Bands:	No visible banding, Slightly visible bands, Clearly visible bands, Prominently visible bands
13	Compensated Brow Ptosis	No signs of brow ptosis, Slight compensated brow ptosis, Moderate compensated brow ptosis, Severe compensated brow ptosis
14	Fat Herniation of Eyelid	No herniation, Slight herniation, Moderate herniation, Severe herniation

**Table 2:** Quantitative facial analysis form with options provided to the MLLMs.

#	Question	Options
1	Can the face be divided into equal vertical fifths?	Yes, No
2	Are the Upper 1/3rd, Middle 1/3rd, and Lower 1/3rd of the face equal?	Yes, No
3	Is the length of an ear equal to the length of the nose?	Yes, No
4	Is the interocular distance equal to nose width?	Yes, No
5	Is the interocular distance equal to the width of one eye (right or left eye fissure width)?	Yes, No
6	Is the mouth width 1.5 times the nose width?	Yes, No
7	Is the face width equal to 4 times the nose width?	Yes, No
8	Can the lower face be divided into equal thirds?	Yes, No
9	Is the width of the face at the malar level equal to the distance from the brows to the menton?	Yes, No
10	The length of the face is 1.618 times the width of the face?	Ratio significantly less (Ratio less than 1.46), Ratio slightly less (Ratio 1.46–1.53), Ratio is ideal (Approximately 1.618), Ratio slightly more (Ratio 1.68–1.77), Ratio significantly more (Ratio greater than 1.77)
11	Ear length to nose width ratio	Ratio significantly less (Ratio less than 1.46), Ratio slightly less (Ratio 1.46–1.53), Ratio is ideal (Approximately 1.618), Ratio slightly more (Ratio 1.68–1.77), Ratio significantly more (Ratio greater than 1.77)
12	Mouth width to interocular distance	Ratio significantly less (Ratio less than 1.46), Ratio slightly less (Ratio 1.46–1.53), Ratio is ideal (Approximately 1.618), Ratio slightly more (Ratio 1.68–1.77), Ratio significantly more (Ratio greater than 1.77)
13	Mouth width to nose width	Ratio significantly less (Ratio less than 1.46), Ratio slightly less (Ratio 1.46–1.53), Ratio is ideal (Approximately 1.618), Ratio slightly more (Ratio 1.68–1.77), Ratio significantly more (Ratio greater than 1.77)
14	Lips-chin distance to interocular distance	Ratio significantly less (Ratio less than 1.46), Ratio slightly less (Ratio 1.46–1.53), Ratio is ideal (Approximately 1.618), Ratio slightly more (Ratio 1.68–1.77), Ratio significantly more (Ratio greater than 1.77)
15	Lips-chin distance to nose width	Ratio significantly less (Ratio less than 1.46), Ratio slightly less (Ratio 1.46–1.53), Ratio is ideal (Approximately 1.618), Ratio slightly more (Ratio 1.68–1.77), Ratio significantly more (Ratio greater than 1.77)

**Table 3:** Qualitative analysis-ranked performance metrics in descending order.

Rank	MLLM (Mean Accuracy)	Rank	Question (Mean Accuracy)	Rank	Face (Mean Accuracy)
1	ChatGPT-4o (0.61)	1	3. Volume/Fat Distribution in Cheeks (0.82)	1	4 (0.80)
2	Gemini 1.5 Pro (0.60)	2	11. Extent of Jowls (0.73)	2	2 (0.77)
3	ChatGPT-4 (0.57)	3	12. Visibility of Platysmal Bands (0.73)	3	9 (0.71)
4	Claude 3.5 Sonnet (0.52)	4	7. Balance/Harmony of Face (0.70)	4	7 (0.70)
		5	13. Compensated Brow Ptosis (0.68)	5	14 (0.70)
		6	4. Overall tone of the skin (0.68)	6	15 (0.68)
		7	5. Presence of Rhytids (Wrinkles) (0.65)	7	1 (0.59)
		8	14. Fat Herniation of Eyelid (0.65)	8	11 (0.62)
		9	1. Identify the skin type (0.55)	9	5 (0.64)
		10	10. Depth of nasolabial folds (0.55)	10	10 (0.46)
		11	2. Skin Texture (0.50)	11	3, 12, 13, (0.45)
		12	8. Nose Proportions/Symmetry (0.50)	12	6 (0.36)
		13	9. Symmetry of nasolabial folds (0.27)		
		14	6. Glogau Classification (0.02)		

**Table 4:** Quantitative analysis-ranked performance metrics in descending order.

Ranking	MLLM (Mean Accuracy)	Rank	Question (Mean Accuracy)	Rank	Face (Mean Accuracy)
1	Gemini 1.5 Pro (0.39)	1	9. Malar Width = Brow to Menton Distance (0.75)	1	8 (0.50)
2	Claude 3.5 Sonnet (0.37)	2	8. Lower Face Division into Thirds (0.70)	2	10 (0.47)
3	ChatGPT-4 (0.32)	3	5. Interocular Distance = Eye Fissure Width (0.53)	3	13 (0.43)
4	ChatGPT-4o (0.32)	4	6. Mouth Width = 1.5 × Nose Width (0.52)	4	12 (0.42)
		5	4. Interocular Distance = Nose Width (0.50)	5	7 (0.40)
		6	2. Upper/Middle/Lower Thirds Equality (0.48)	6	1 (0.37)
		7	7. Face Width = 4 × Nose Width (0.48)	7	6 (0.37)
		8	1. Vertical Fifths Equality (0.43)	8	9 (0.35)
		9	3. Ear Length = Nose Length (0.38)	9	5, 11 (0.33)
		10	14. Lips-Chin Distance to Interocular Distance (0.18)	10	3 (0.28)
		11	13. Mouth width to nose width (0.15)	11	2 (0.27)
		12	12. Mouth Width to Interocular Distance (0.12)	12	1, 14, 15 (0.25)
		13	11. Ear Length to Nose Width Ratio (0.020)		
		14	15. Lips-Chin Distance to Nose Width (0.020)		
		15	10. Face Length = 1.618 × Face Width (0.00)		

## Data Availability

The original contributions presented in this study are included in the article/Supplementary Materials. Further inquiries can be directed to the corresponding author.

## Supplementary Materials

The following supporting information can be downloaded at: https://www.mdpi.com/article/10.3390/jcm14103484/s1. Table S1: Qualitative analysis mean scores and standard deviations for each question by LLM. Table S2: Quantitative analysis mean scores and standard deviations for each question by LLM. Table S3: (a) Kappa coefficients by comparing AI models overall general face evaluation to the plastic surgeons’ rating; (b) Kappa coefficients by comparing AI models ratio evaluation to the manual measurements.

## Abbreviations

The following abbreviations are used in this manuscript:

AI	Artificial Intelligence
MLLM	Multimodal Large Language Model
GAN	Generative Adversarial Network
ICC	Intraclass Correlation Coefficient
ANOVA	Analysis of Variance
PHI	Protected Health Information
