# Supplementary material for: Facial Analysis for Plastic Surgery in the Era of Artificial Intelligence: A Comparative Evaluation of Multimodal Large Language Models
Source: J Clin Med. 2025 May 16;14(10):3484. doi: 10.3390/jcm14103484 (PMC12112453; doi:10.3390/jcm14103484)
Supplement: Supplementary file 1 [file jcm-14-03484-s001.zip › jcm-3607219-supplementary.pdf]

SUPPLEMENTAL FILES:

Table S1: Qualitative Analysis Mean Scores and Standard Deviations for Each Question by LLM.

#	Question	LLM	Mean	Std. Deviation
1	Identify the skin type	ChatGPT-4	0.60	0.507
		ChatGPT-4o	0.67	0.488
		Claude 3.5 Sonnet	0.20	0.414
		Gemini 1.5 Pro	0.73	0.458
		Total	0.55	0.502
2	Comment on the skin texture	ChatGPT-4	0.67	0.488
		ChatGPT-4o	0.47	0.516
		Claude 3.5 Sonnet	0.40	0.507
		Gemini 1.5 Pro	0.47	0.516
		Total	0.50	0.504
3	Volume and Fat Distribution in Cheeks	ChatGPT-4	0.80	0.414
		ChatGPT-4o	0.73	0.458
		Claude 3.5 Sonnet	0.80	0.414
		Gemini 1.5 Pro	0.93	0.258
		Total	0.82	0.390
4	How would you rate the overall tone of the skin?	ChatGPT-4	0.60	0.507
		ChatGPT-4o	0.87	0.352
		Claude 3.5 Sonnet	0.67	0.488
		Gemini 1.5 Pro	0.60	0.507
		Total	0.68	0.469
5	Presence of Rhytids (Wrinkles) in the face:	ChatGPT-4	0.67	0.488
		ChatGPT-4o	0.60	0.507
		Claude 3.5 Sonnet	0.67	0.488
		Gemini 1.5 Pro	0.67	0.488
		Total	0.65	0.481
6		ChatGPT-4	0.00	0.000

	Using the Glogau classification, determine the degree of photoaging	ChatGPT-4o	0.07	0.258
		Claude 3.5 Sonnet	0.00	0.000
		Gemini 1.5 Pro	0.00	0.000
		Total	0.02	0.129
7	Assess the Overall balance and harmony of the face.	ChatGPT-4	0.73	0.458
		ChatGPT-4o	0.73	0.458
		Claude 3.5 Sonnet	0.60	0.507
		Gemini 1.5 Pro	0.73	0.458
		Total	0.70	0.462
8	Overall Proportions and Symmetry of the Nose	ChatGPT-4	0.47	0.516
		ChatGPT-4o	0.47	0.516
		Claude 3.5 Sonnet	0.47	0.516
		Gemini 1.5 Pro	0.60	0.507
		Total	0.50	0.504
9	Assess the symmetry of the nasolabial folds	ChatGPT-4	0.20	0.414
		ChatGPT-4o	0.33	0.488
		Claude 3.5 Sonnet	0.33	0.488
		Gemini 1.5 Pro	0.20	0.414
		Total	0.27	0.446
10	Assess the depth of the nasolabial folds	ChatGPT-4	0.73	0.458
		ChatGPT-4o	0.47	0.516
		Claude 3.5 Sonnet	0.53	0.516
		Gemini 1.5 Pro	0.47	0.516
		Total	0.55	0.502
11	Extent of Jowls	ChatGPT-4	0.67	0.488
		ChatGPT-4o	0.67	0.488
		Claude 3.5 Sonnet	0.87	0.352
		Gemini 1.5 Pro	0.73	0.458
		Total	0.73	0.446
12	Visibility of Platysmal Bands:	ChatGPT-4	0.60	0.507

		ChatGPT-4o	0.87	0.352
		Claude 3.5 Sonnet	0.73	0.458
		Gemini 1.5 Pro	0.73	0.458
		Total	0.73	0.446
13	Compensated Brow Ptosis.	ChatGPT-4	0.60	0.507
		ChatGPT-4o	0.73	0.458
		Claude 3.5 Sonn	0.67	0.488
		Gemini 1.5 Pro	0.73	0.458
		Total	0.68	0.469
14	Fat Herniation of Eyelid.	ChatGPT-4	0.67	0.488
		ChatGPT-4o	0.87	0.352
		Claude 3.5 Sonn	0.33	0.488
		Gemini 1.5 Pro	0.73	0.458
		Total	0.65	0.481
	Total	ChatGPT-4	0.57	0.496
		ChatGPT-4o	0.61	0.489
		Claude 3.5 Sonn	0.52	0.501
		Gemini 1.5 Pro	0.60	0.492
		Total	0.57	0.495

Supplemental Table S2: Quantitative Analysis Mean Scores and Standard Deviations for Each Question by LLM.

#	Question	LLM	Mean	Std. Deviation
1	Can the face be divided into equal vertical fifths?	ChatGPT-4	0.47	0.516
		ChatGPT-4o	0.33	0.488
		Claude 3.5 Sonnet	0.47	0.516
		Gemini 1.5 Pro	0.47	0.516
		Total	0.43	0.500
2	Are the Upper 1/3rd, Middle 1/3rd, and Lower 1/3rd of the face equal?	ChatGPT-4	0.27	0.458
		ChatGPT-4o	0.60	0.507
		Claude 3.5 Sonnet	0.47	0.516
		Gemini 1.5 Pro	0.60	0.507
		Total	0.48	0.504
3	Is the length of an ear equal to the length of the nose?	ChatGPT-4	0.40	0.507
		ChatGPT-4o	0.53	0.516
		Claude 3.5 Sonnet	0.33	0.488
		Gemini 1.5 Pro	0.27	0.458
		Total	0.38	0.490
4	Is the interocular distance equal to nose width?	ChatGPT-4	0.33	0.488
		ChatGPT-4o	0.60	0.507
		Claude 3.5 Sonnet	0.20	0.414
		Gemini 1.5 Pro	0.87	0.352
		Total	0.50	0.504
5	Is the interocular distance equal to the width of one eye (right or left eye fissure width)?	ChatGPT-4	0.40	0.507
		ChatGPT-4o	0.53	0.516
		Claude 3.5 Sonnet	0.67	0.488
		Gemini 1.5 Pro	0.53	0.516
		Total	0.53	0.503
6	Is the mouth width 1.5 times the nose width?	ChatGPT-4	0.53	0.516

		ChatGPT-4o	0.13	0.352
		Claude 3.5 Sonnet	0.93	0.258
		Gemini 1.5 Pro	0.47	0.516
		Total	0.52	0.504
7	Is the face width equal to 4 times the nose width?	ChatGPT-4	0.53	0.516
		ChatGPT-4o	0.40	0.507
		Claude 3.5 Sonnet	0.60	0.507
		Gemini 1.5 Pro	0.40	0.507
		Total	0.48	0.504
8	Can the lower face be divided into equal thirds?	ChatGPT-4	0.60	0.507
		ChatGPT-4o	0.47	0.516
		Claude 3.5 Sonnet	0.80	0.414
		Gemini 1.5 Pro	0.93	0.258
		Total	0.70	0.462
9	Is the width of the face at the malar level equal to the distance from the brows to the menton?	ChatGPT-4	0.67	0.488
		ChatGPT-4o	0.80	0.414
		Claude 3.5 Sonnet	0.73	0.458
		Gemini 1.5 Pro	0.80	0.414
		Total	0.75	0.437
10	The length of the face is 1.618 times the width of the face?	ChatGPT-4	0.00	0.000
		ChatGPT-4o	0.00	0.000
		Claude 3.5 Sonnet	0.00	0.000
		Gemini 1.5 Pro	0.00	0.000
		Total	0.00	0.000
11	Ear length to nose width ratio	ChatGPT-4	0.00	0.000
		ChatGPT-4o	0.00	0.000
		Claude 3.5 Sonnet	0.00	0.000
		Gemini 1.5 Pro	0.07	0.258
		Total	0.02	0.129
12	Mouth width to interocular distance	ChatGPT-4	0.20	0.414

		ChatGPT-4o	0.07	0.258
		Claude 3.5 Sonnet	0.13	0.352
		Gemini 1.5 Pro	0.07	0.258
		Total	0.12	0.324
13	Mouth width to nose width	ChatGPT-4	0.13	0.352
		ChatGPT-4o	0.13	0.352
		Claude 3.5 Sonnet	0.20	0.414
		Gemini 1.5 Pro	0.13	0.352
		Total	0.15	0.360
14	Lips-chin distance to interocular distance	ChatGPT-4	0.20	0.414
		ChatGPT-4o	0.20	0.414
		Claude 3.5 Sonnet	0.07	0.258
		Gemini 1.5 Pro	0.27	0.458
		Total	0.18	0.390
15	Lips-chin distance to nose width	ChatGPT-4	0.07	0.258
		ChatGPT-4o	0.00	0.000
		Claude 3.5 Sonnet	0.00	0.000
		Gemini 1.5 Pro	0.00	0.000
		Total	0.02	0.129
	Total	ChatGPT-4	0.32	0.468
		ChatGPT-4o	0.32	0.468
		Claude 3.5 Sonnet	0.37	0.485
		Gemini 1.5 Pro	0.39	0.489
		Total	0.35	0.478

#	**Question**	ChatGPT-4o<br>Mean (SD)	ChatGPT-4<br>Mean (SD)	Claude 3.5 Sonnet<br>Mean (SD)	Gemini 1.5 Pro<br>Mean (SD)
1	Can the face be divided into equal vertical fifths?	0.33 ± 0.49	0.47 ± 0.52	0.47 ± 0.52	0.47 ± 0.52
2	Are the Upper 1/3rd, Middle 1/3rd, and Lower 1/3rd of the face equal?	0.60 ± 0.51	0.27 ± 0.46	0.47 ± 0.52	0.53 ± 0.52

3	Is the length of an ear equal to the length of the nose?	0.53 ± 0.52	0.40 ± 0.51	0.33 ± 0.49	0.27 ± 0.46
4	Is the interocular distance equal to nose width?	0.60 ± 0.51	0.33 ± 0.49	0.20 ± 0.41	0.73 ± 0.46
5	Is the interocular distance equal to the width of one eye (right or left eye fissure width)?	0.53 ± 0.52	0.40 ± 0.51	0.67 ± 0.49	0.47 ± 0.52
6	Is the mouth width 1.5 times the nose width?	0.33 ± 0.49	0.47 ± 0.52	0.60 ± 0.51	0.53 ± 0.52
7	Is the face width equal to 4 times the nose width?	0.93 ± 0.26	0.53 ± 0.52	0.07 ± 0.26	0.93 ± 0.26
8	Can the lower face be divided into equal thirds?	0.60 ± 0.51	0.60 ± 0.51	0.67 ± 0.49	0.73 ± 0.46
9	Is the width of the face at the malar level equal to the distance from the brows to the menton?	0.13 ± 0.35	0.40 ± 0.51	0.20 ± 0.41	0.00 ± 0.00
10	The length of the face is 1.618 times the width of the face?	0.00 ± 0.00	0.00 ± 0.00	0.00 ± 0.00	0.07 ± 0.26
11	Ear length to nose width ratio	0.07 ± 0.26	0.07 ± 0.26	0.07 ± 0.26	0.00 ± 0.00
12	Mouth width to interocular distance	0.07 ± 0.26	0.07 ± 0.26	0.07 ± 0.26	0.27 ± 0.46
13	Mouth width to nose width	0.07 ± 0.26	0.07 ± 0.26	0.00 ± 0.00	0.00 ± 0.00
14	Lips-chin distance to interocular distance	0.20 ± 0.41	0.20 ± 0.41	0.07 ± 0.26	0.27 ± 0.46
15	Lips-chin distance to nose width	0.00 ± 0.00	0.07 ± 0.26	0.00 ± 0.00	0.00 ± 0.00

Supplemental Table S3. (a). Kappa coefficients by comparing AI models overall general face evaluation to the plastic surgeons' rating; (b) Kappa coefficients by comparing AI models ratio evaluation to the manual measurements.

(a)				
**Question**	**CG4.kappa**	**CG4o.kappa**	**CL.kappa**	**GMN.kappa**
Identify the skin type	0.224	0.185	0.000	0.211
Comment on the skin texture	0.390	0.048	0.000	0.091
Volume and Fat Distribution in Cheeks	0.737	0.565	0.286	0.894

How would you rate the overall tone of the skin?	0.211	0.773	0.000	-0.071
Presence of Rhytids (Wrinkles) in the face:	0.813	0.674	0.525	0.798
Using the Glogau classification, determine the degree of photoaging	0.000	-0.024	0.020	0.020
Assess the Overall balance and harmony of the face.	0.792	0.739	0.526	0.811
Overall Proportions and Symmetry of the Nose	0.068	-0.193	-0.193	0.000
Assess the symmetry of the nasolabial folds	0.419	0.226	0.118	0.000
Assess the depth of the nasolabial folds	0.750	0.382	0.426	0.610
Extent of Jowls	0.566	0.312	1.000	0.577
Visibility of Platysmal Bands:	-0.098	-0.071	-0.111	-0.082
Compensated Brow Ptosis	0.426	0.370	0.426	0.370
Fat Herniation of Eyelid	0.000	0.000	0.000	0.000
Cohen's Kappa was calculated for Q1 and weighted Kappa was calculated for Q2-Q14				
**(b)**				
**Question**	**CG4.kappa**	**CG4o.kappa**	**CL.kappa**	**GMN.kappa**
Can the face be divided into equal vertical fifths?	-0.008	-0.282	-0.043	-0.121

Are the Upper 1/3rd, Middle 1/3rd, and Lower 1/3rd of the face equal?	-0.410	0.224	0.000	0.118
Is the length of an ear equal to the length of the nose?	-0.047	0.167	0.020	0.000
Is the interocular distance equal to nose width?	-0.071	0.062	0.022	-0.111
Is the interocular distance equal to the width of one eye (right or left eye fissure width)?	-0.174	-0.105	0.000	0.143
Is the mouth width 1.5 times the nose width?	-0.053	-0.136	0.000	0.103
Is the face width equal to 4 times the nose width?	-0.129	0.000	0.000	0.000
Can the lower face be divided into equal thirds?	0.167	0.286	-0.190	-0.111
Is the width of the face at the malar level equal to the distance from the brows to the menton?	0.000	0.000	0.000	0.000
The length of the face is 1.618 times the width of the face?	0.019	0.058	0.002	-0.008

Ear length to nose width ratio	0.013	-0.002	0.029	0.047
Mouth width to interocular distance	-0.082	-0.050	0.000	-0.163
Mouth width to nose width	-0.026	-0.021	-0.008	0.020
Lips-chin distance to interocular distance	-0.053	-0.136	0.040	0.000
Lips-chin distance to nose width	0.000	0.000	0.000	0.000
Cohen's Kappa was calculated for Q1-Q9, and weighted Kappa was calculated for Q10-Q15				
